# A Novel Tandem Reporter Quantifies RNA Polymerase II Termination in Mammalian Cells

**DOI:** 10.1371/journal.pone.0006193

**Published:** 2009-07-09

**Authors:** Ayan Banerjee, Mimi C. Sammarco, Scott Ditch, Jeffrey Wang, Ed Grabczyk

**Affiliations:** The Department of Genetics, Louisiana State University Health Sciences Center, New Orleans, Louisiana, United States of America; New Mexico State University, United States of America

## Abstract

**Background:**

Making the correct choice between transcription elongation and transcription termination is essential to the function of RNA polymerase II, and fundamental to gene expression. This choice can be influenced by factors modifying the transcription complex, factors modifying chromatin, or signals mediated by the template or transcript. To aid in the study of transcription elongation and termination we have developed a transcription elongation reporter system that consists of tandem luciferase reporters flanking a test sequence of interest. The ratio of expression from the reporters provides a measure of the relative rates of successful elongation through the intervening sequence.

**Methodology/Principal Findings:**

Size matched fragments containing the polyadenylation signal of the human β-actin gene (ACTB) and the human β-globin gene (HBB) were evaluated for transcription termination using this new ratiometric tandem reporter assay. Constructs bearing just 200 base pairs on either side of the consensus poly(A) addition site terminated 98% and 86% of transcription for ACTB and HBB sequences, respectively. The nearly 10-fold difference in read-through transcription between the two short poly(A) regions was eclipsed when additional downstream poly(A) sequence was included for each gene. Both poly(A) regions proved very effective at termination when 1100 base pairs were included, stopping 99.6% of transcription. To determine if part of the increased termination was simply due to the increased template length, we inserted several kilobases of heterologous coding sequence downstream of each poly(A) region test fragment. Unexpectedly, the additional length reduced the effectiveness of termination of HBB sequences 2-fold and of ACTB sequences 3- to 5-fold.

**Conclusions/Significance:**

The tandem construct provides a sensitive measure of transcription termination in human cells. Decreased Xrn2 or Senataxin levels produced only a modest release from termination. Our data support overlap in allosteric and torpedo mechanisms of transcription termination and suggest that efficient termination is ensured by redundancy.

## Introduction

While it is unclear precisely what percentage of human RNA polymerases initiate and successfully complete gene transcription, live cell imaging has been used to estimate that it is on the order of 1% [1]. The RNA polymerase II transcription complex may be influenced by factors modifying the transcription complex, factors modifying chromatin, or intrinsic properties of the template and transcript that affect the constant choice between transcription elongation and transcription termination [2]–[4]. Most of the abortive transcription takes place near the promoter. However, once RNA polymerase II escapes from the promoter region of a gene, it shifts into a highly processive mode in which it can actively transcribe the DNA template for megabases.

An elongating polymerase must maintain a balance between being too readily terminated such that it cannot reach the end of a gene, and being an unstoppable juggernaut. Continued transcription past the end of a gene is not desirable. When an RNAP II molecule in the cell engages in such nonproductive elongation, it not only wastes energy but also risks interfering with the expression of neighboring genes [5]. More importantly, evidence is accumulating that termination is required for efficient mRNA processing and export, and therefore, for protein expression. Proper termination has been linked to mRNA 3′-end processing and mRNP export through the nuclear pore in yeast cells [6]. It has also recently been suggested that transcriptional termination is linked to mRNA processing and may be required for optimal gene expression in human cells [7]. Consequently, multiple mechanisms have evolved to terminate transcription efficiently at the ends of genes.

Currently, there are several models for polyadenylation associated transcription termination; the allosteric model, the torpedo model, and the combined allosteric/torpedo model. The original formulation of the allosteric, or anti-terminator model, posits that polyadenylation signals cause a change in the complement of anti-termination factors associated with RNAP II, making it more susceptible to termination [8]. Whereas, an early formulation of the torpedo model suggests that a 5′ to 3′ exonuclease attacks the transcript at the poly(A) cleavage point and degrades the nascent transcript in pursuit of the RNA polymerase, leading to termination [9]. More recently, groups have presented evidence for a combined allosteric/torpedo model [10], as well as a role for the phosphorylation state of the RNAP II C-terminal domain (CTD) in termination and 3′ end formation [11].

Transcription termination is an important, yet under-studied aspect of gene regulation. In many cases, transcription elongation remains a rate-limiting step in gene expression. To some degree, the current shortage of information on transcription termination reflects the difficulties involved in its study. The classic standard of transcription elongation and termination research has been the nuclear run-on assay (NRO), in which engaged RNAP II complexes in prepared nuclei incorporate a radiolabeled rNTP as they “run-on” for some distance during incubation *in vitro*. RNA labeled by RNAP II in this way is then hybridized to specific targets to assess the relative level of actively transcribing RNA polymerases within those regions at the time the nuclei were prepared [12], [13]. However, the NRO assay is cumbersome and not overly sensitive, requiring on the order of 2×10^7^ cells per sample and a strong promoter to work. Most single copy mammalian genes do not have expression levels sufficient to produce reliable signals [14]. Consequently, much of the NRO analysis performed to analyze transcription termination in mammalian cells has been done using transiently transfected plasmids, rather than native genes in chromatin. While a great deal can be learned from transient assays, the role of epigenetic chromatin modification in termination, for example, cannot be fully explored. Conversely, while chromatin immunoprecipitation (ChIP) using anti-RNAP II antibodies can show association of RNAP II with a particular DNA region in native chromatin, ChIP cannot determine whether the polymerase was actively transcribing.

In short, transcription termination is an important step in gene expression, but can be difficult to measure in mammalian cells. To address this difficulty, we built a novel tandem reporter construct capable of assaying transcription termination from a single copy integrated into a human chromosome. The ratio of expression from the downstream reporter compared to the control reporter upstream provides a measure of the relative rates of successful elongation through the intervening sequence. Here we use this system to measure the efficiency of termination orchestrated by multiple elements contained within fragments of the human β-actin (ACTB) and human β-globin (HBB) terminator regions. We find that our system provides a sensitive ratiometric measure of transcription termination in live cells compatible with high throughput approaches.

We sought to probe the relative contributions of the torpedo and allosteric models of transcription termination using this system. Our system did not support a dominant role for any model or factor tested, such as XRN2 or Senataxin, in termination mediated by HBB or ACTB sequences. Rather, our findings suggest a cooperative effect among multiple elements contribute to termination, with efficiency mediated by redundancy.

## Results and Discussion

### A tandem reporter quantifies transcription elongation

**Figure 1:**
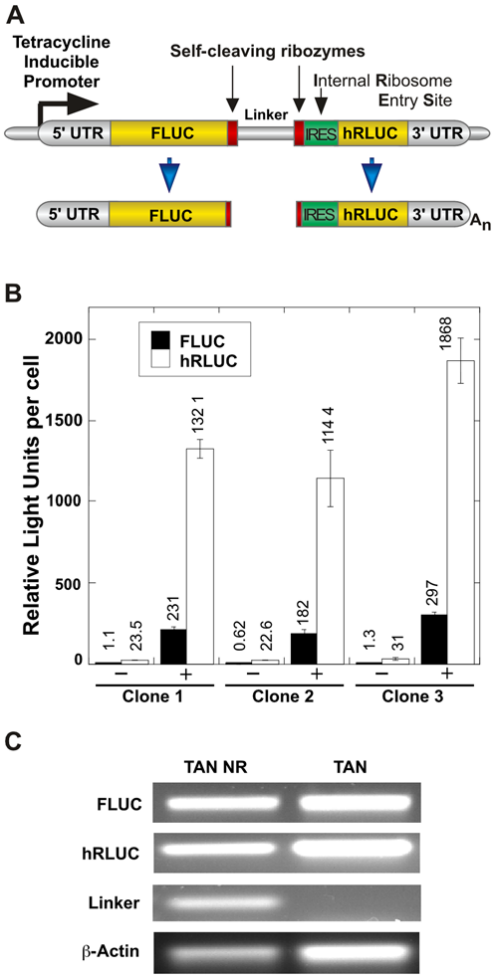
The use of tandem reporters to measure transcription elongation. *A*, A tetracycline-regulated promoter drives transcription through the tandem reporters. Self-cleaving hammerhead ribozymes cut the RNA transcript, separating both FLUC and hRLUC expressing RNA fragments from the test sequence. An internal ribosome entry sequence (IRES) enhances translation of the uncapped hRLUC expression fragment by replacing functions of the 5′ cap and untranslated region (5′ UTR). The relative ratio between the reporters measures the transcriptional impediment presented by an insert. The 5′ reporter (FLUC) is firefly luciferase and the 3′ reporter (hRLUC) is a humanized sea pansy luciferase. If the RNA polymerase terminates transcription in the inserted test sequence, the 3′ reporter will not be transcribed. *B*, Robust, reproducible induction of FLUC and hRLUC activities in clonal cell lines. Graphs display the mean luciferase activity per cell in Relative Light Units (RLU) for FLUC and hRLUC from clonal cell lines containing a single integrated copy of a control tandem reporter construct. Cells were cultured without (−) or with (+) the addition of doxycycline for 24 hours to induce transcription from the promoter. Extracts representing 15,000 cells were assayed for luciferase activity. The mean induction for FLUC expression was 237±29 fold, and 56±3 for hRLUC. Error bars indicate the S.E.M. for a sample number of three. *C*, Ribozymes self-cleave in a human cell line. Gel shows yield of RT-PCR products Transcription was induced with doxycycline and RNA was harvested after 24 hours. RT-PCR was conducted using primers designed for FLUC and hRLUC coding regions, and primers designed to span the ribozymes and the 275 bp of linker sequence between the two ribozymes. TAN-NR does not contain self-cleaving ribozymes, and all three sets of primers successfully amplify the target. β-actin primers were included as an amplification control. RT-PCR products were separated on a 1% agarose gel.

We designed a tandem reporter construct to measure the rate of transcription termination in living cells. Our goal was to express two quantifiable reporters in tandem from an inducible promoter in a construct located within a single, unique chromosomal location. Transcription initiates at a tetracycline-inducible promoter, proceeds through the first reporter (FLUC), and then must traverse the test termination sequence before reaching the second reporter (hRLUC) (Figure 1A). The 1.7 kb FLUC coding region in the first part of the transcription unit means that the transcribing polymerase is well past promoter escape and into processive elongation before it encounters the test sequence. Furthermore, the inducible promoter and tandem vector design ensure that expression of both control and test reporters are the result of the same polymerase, and that the ratio of the two reporters reflects termination mediated by the center fragment. Self-cleaving ribozymes flank the test sequence, so that the transcribed termination sequence does not become part of either reporter mRNA fragment. Therefore, the sequences of the reporter mRNA fragments are independent from the test sequence inserted between the ribozymes (Figure 1A, below large arrows).

The first reporter mRNA fragment contains firefly luciferase (FLUC) and has a 5′ cap but lacks a polyadenylation signal and hence, a poly(A) tail. An A_32_ tract just 5′ of the self-cleaving ribozyme aids in translation of the 5′ FLUC expression cassette (Sammarco & Grabczyk, unpublished). FLUC functions as the control reporter and is expressed independently of the inserted sequence. The downstream hRLUC reporter lacks a 5′ cap but translation is aided by the presence of an internal ribosome entry site (IRES). Because a chromosomal location may more closely represent a native gene than would an episome or transient transfections, we adopted the Invitrogen Flp-In™ T-REx system. The constructs were introduced by the site-specific Flp recombinase into a single genomic location to make stable cell lines. The chromosomal location and orientation is consistent for all tested inserts, removing a potentially significant variable.

The constitutive expression of the tet-repressor in the T-REx HEK 293 cell line represses the construct promoter in the absence of tetracycline. To test the ability of a single integrated copy of the tandem construct to express both reporter genes simultaneously and reproducibly upon induction, extracts representing 15,000 cells from three independently isolated clonal lines bearing tandem reporters were assayed for luciferase activities (Figure 1B). Extracts were prepared from cells maintained in normal growth media (−) or were treated with 1 µg/mL doxycycline (+), and harvested 24 hours after induction. Cell lines with a single integrated tandem construct showed strong expression of both FLUC and hRLUC reporters upon induction (compare minus and plus induction in Figure 1B). The mean induction for FLUC expression was 237±29 fold, and 56±3 for hRLUC. The lower hRLUC induction reflects a higher background for hRLUC expression due to the nature of the hRLUC cassette, which is essentially a transcription trap. Whereas, the FLUC RNA fragment has cap-dependent translation, and must initiate at or near the inducible start site to be translated efficiently, the combination of the ribozyme and the IRES allow translation of the hRLUC fragment even if transcription starts far upstream of the inducible start site. This background is not generally a problem because hRLUC expression was induced over 50-fold upon de-repression of the promoter. Thus, we can be confident that in the induced state, at least 98% of the transcribing polymerases initiated at the promoter and traversed FLUC and the intervening sequences before transcribing hRLUC. The mean induced FLUC and hRLUC activity was similar between individual cell lines (see Figure 1B), and we suspect that much of the variability that we did see was due to variability in counting and plating cells. More importantly for our purposes, the ratio of hRLUC to FLUC luminescence was very reproducible from clone to clone. This ratio is calculated for each well from sequential reads of the two luciferase values, and is independent of cell number. Clonal cell lines 1, 2 and 3 had hRLUC/FLUC ratios of 6.19, 6.27 and 6.28, respectively, which were not significantly different (p>0.05).

The self-cleaving hammerhead ribozyme used in these constructs has been shown to cleave itself efficiently during *in vitro* transcription reactions by our group and others [15]–[17]. Since the ribozymes contribute to the effectiveness of the tandem construct, it was necessary to confirm ribozyme cleavage in the cell. Ribozyme self-cleavage was verified by PCR amplification of reverse-transcribed RNA prepared from cell lines. Primers designed to span the ribozymes yielded little or no PCR product, demonstrating that the mRNA produced by the product was successfully cleaved by the ribozymes, and thus generated little or no cDNA during reverse transcription (Figure 1C). PCR amplification with the same primers on a construct similar to the tandem construct but lacking ribozymes (TAN NR) yielded a PCR product (Figure 1C). β-actin was chosen as an endogenous gene to control for RNA loading (Figure 1C). These results indicate that ribozymes in the tandem constructs cleave efficiently, and that the luciferase reporters are expressed from the fragmented mRNA in sufficient quantity for use in a 96-well high throughput format.

### Poly(A) associated termination is directly correlated with downstream flanking DNA

**Figure 2:**
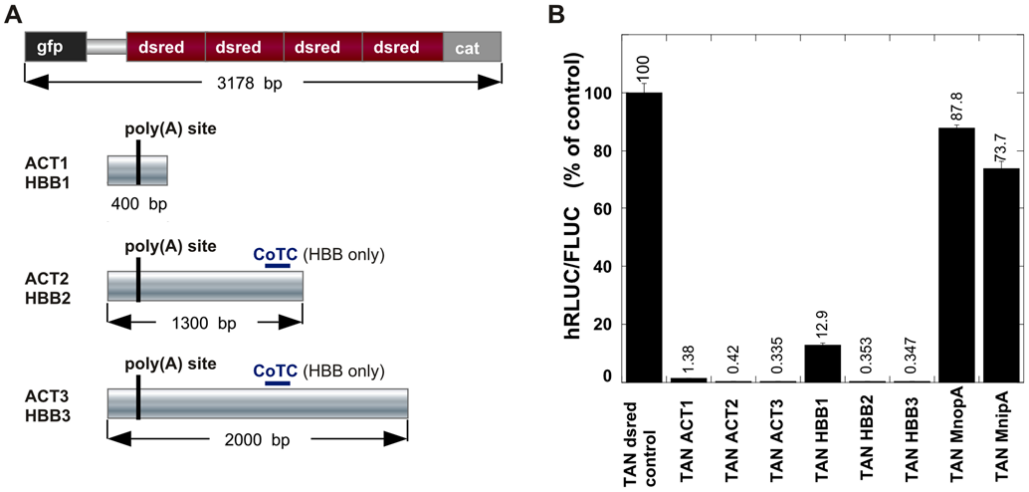
Actin and globin 3′ sequences provide robust transcription termination. *A*, Schematic maps of DNA inserts used with the tandem vector indicating relative size. Fragments of green fluorescent protein, red fluorescent protein and chloramphenicol acetyl transferase coding regions are not full-length, and do not make functional protein. The polyadenylation regions used in the experiments are shown below, drawn to scale. The position of the poly(A) addition site is indicated by a vertical black bar. The mini actin polyadenylation insert (MnipA) would be about the width of that bar. *B*, Graph showing ratios of expressed hRLUC to FLUC activities for the indicated constructs in stable cell lines. Inclusion of 400 to approximately 2000 bp of sequence containing either the actin (ACT1-3) or globin (HBB1-3) poly(A) addition site effectively terminated transcription elongation. Transcription was induced with doxycycline and cells were harvested after 24 hours. Results for each cell line are expressed as a percentage of the dsred control cell line, which does not contain a polyadenylation sequence. Longer poly(A) region fragments that included a putative co-transcription cleavage element (CoTC) were included in the globin constructs, and reduced read-through expression of the downstream reporter to 1–2% of the control dsred cell line for all constructs. A minimal poly(A) signal sequence provides modest transcription termination (MnipA). The control (MnopA) contained a nearly identical sequence except for two changed bases in the hexamer part of the polyadenylation signal. The y axis values are hRLUC/FLUC expression ratios normalized to a positive cell lysate run in each plate. All of the changes are significant (p<0.05) when compared to the dsred control. Error bars indicate the S.E.M. for a sample number of three.

To test the effectiveness of our system in measuring transcription termination, we chose to use two well-characterized polyadenylation/termination regions of human origin. The relationship between transcription termination and polyadenylation has been well established in both the human β-globin (HBB) gene [18] and the human β-actin (ACTB) gene [19]. We used our construct to quantify transcription termination in defined sections of polyadenylation sequence from both HBB and ACTB (Figure 2A). As a control, we used a tandem vector containing partial coding regions of red fluorescent protein (dsred). This widely used reporter gene's coding region is unlikely to contain functioning termination elements, and furthermore, also unlikely to contain functioning promoter elements that could contribute to background transcription. We compared the empty TAN0 control construct to tandem constructs containing either a single, double or tetrameric dsred fragment and found no difference in transcription elongation (not shown). We used the longest template in the dsred series as our control, as it exhibited little termination yet was over a kilobase longer than our longest test templates (Figure 2A, top).

All the ACTB constructs showed a robust degree of transcription termination in our system. The TAN ACT1 construct contained only 400 bp of the ACTB poly(A) region, yet terminated over 98% of transcribing polymerases, as measured by the change in hRLUC/FLUC expression ratio compared to the tandem construct with the dsred tetramer control insert (Figure 2B). The ACTB constructs demonstrated increased transcription termination as more sequence downstream of the poly(A) addition site was included in the construct. ACT2 was approximately 3-fold more effective than ACT1, and ACT3 was 4-fold more effective (Figure 2B).

Analysis of the human HBB poly(A) region showed a similar trend with increased termination correlating with increased sequence length (Figure 2B). When the ACT1 sequence is compared to the HBB1 sequence, there is nearly a 10-fold difference in transcription read-through, indicating that the ACTB sequence terminates elongation more effectively within a shorter sequence length. However, HBB2 has an additional 1100 base pairs of *HBB* gene sequence that includes a putative co-transcriptional cleavage (CoTC) site [20] and terminates transcription about 36 times more effectively than HBB1. Termination by HBB2 is at least as effective as that of the like-sized ACT2. Further addition of downstream sequences in HBB3 does not greatly increase the degree of termination. This suggests that either we have included the majority of termination signals in a little over a kilobase downstream of the poly(A) site, or that we have reached the limit of detection in our system. Similarly, we see no significant difference in transcription termination between the strongest terminators we tested in the tandem vectors, ACT3 and HBB3. Consequently, because we have no reason to believe that the longest termination sequences we tested from two different genes would provide precisely the same degree of termination, we conclude that 99.6% termination is about the limit of detection in our system.

Since the shortest ACTB sequence that we tested, TAN ACT1, was very effective at terminating transcription elongation in just a few hundred basepairs, we decided to test a minimal polyadenylation signal (MnipA) derived from the core of ACTB. The 59 base-pair sequence contained the AATAAA hexamer signal, the poly(A) addition site and the most proximal downstream GT-rich tract. The MnipA sequence showed a 25% decrease from the TAN dsred control construct (Figure 2B). MnopA, is identical to MnipA except for two base pairs in the hexamer sequence (AATATT instead of AATAAA). Surprisingly, MnopA retained some ability to terminate as the construct showed a modest decrease (12%) relative to the control (Figure 2B). Given the statistical probability of occurrence of AATAAA hexamers and GT-rich tracts in a genome, the modest termination afforded by the minimal poly(A) region itself is not that surprising, and supports the hypothesis that multiple signals are needed for strong termination to prevent otherwise small mutations from truncating transcription units.

### The role of Xrn2 and Senataxin in transcription termination

The ‘torpedo’ model of transcription termination, predicts that an exonuclease or helicase enters the nascent transcript at the poly(A) cleavage point and contributes to termination [9]. A 5′ to 3′ exonuclease, (Rat1p in yeast, Xrn2 in humans) that contributes to RNAP II termination and 3′ end formation has been the focus of much study [21], [22]. The model proposes that Xrn2 loads on the nascent transcript at the poly(A) cleavage site, or on the free end generated by CoTC cleavage in the case of HBB termination, and degrades the nascent transcript, eventually catching the RNA polymerase, which then releases the template [21], [22]. Similarly, recent studies have also implicated the helicase senataxin (sen1p in yeast) in transcription termination [23].

It has been demonstrated that the yeast homologue Rat1p prefers RNA with a 5′ monophosphate as a substrate, which explains why intact, capped messages are not a target [24]. Furthermore, Rat1p activity on substrates with a 5′ OH or structured 5′ end is also greatly reduced relative to its preferred substrate [25]. The self-cleaving ribozymes we employ not only leave a 5′ OH upon cleavage, but also have a structured end. If Xrn2 has the same preference as its yeast homologue, Rat1p, then our ribozyme-containing construct should allow us an opportunity to short circuit Xrn2 activity. If ribozyme cleavage precedes the arrival of Xrn2, the transcribing polymerase would escape the chase-down and hRLUC will be expressed. Consequently, it was possible that part of the length-dependent effect we saw in Figure 2B was due to ribozyme cleavage short-circuiting the torpedo model. To determine whether this was the case, we added spacer DNA between the ribozymes and the poly(A) signals by cloning the poly(A) regions within a stretch of polylinker remaining in the TAN dsred control vector (see Figure 2A, top).

**Figure 3:**
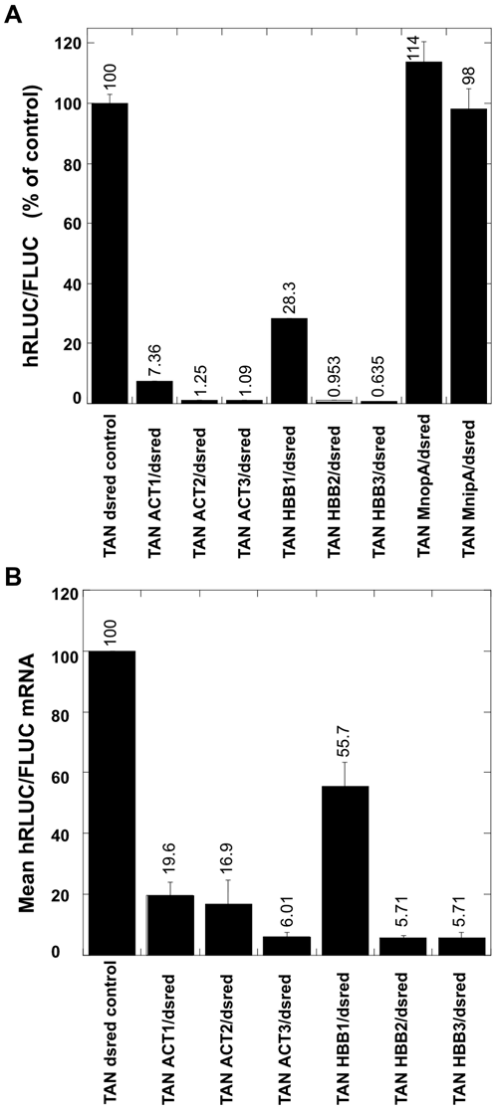
Runner sequence offers modest release from transcription termination. *A*, The inclusion of 2.6 kilobases of dsred tetrameric sequence downstream (3′) to the ACT and HBB sequences provides modest release from transcription termination. Transcription was induced with doxycycline and cells harvested after 24 hours. The y-axis values are hRLUC/FLUC expression ratios normalized to a positive cell lysate run in each plate and expressed as a percentage of the dsred control cell line. All of the changes are significant (p<0.05) when compared to the dsred control. The error bars indicate the S.E.M. for a sample number of three. *B*, Real-time RT-PCR analysis. FLUC and hRLUC mRNA was measured by real-time RT-PCR and expressed as the relative ratio of hRLUC/FLUC mRNA. Results are shown as a percentage of the dsred control cell line. The ratio decreased as larger tracts of polyadenylation sequence are included in the tandem construct. Error bars indicate the S.E.M. for a sample number of three.

Surprisingly, the presence of dsred sequence 3′ to the poly(A) regions decreased the effectiveness of transcription termination in all tested constructs (Figure 3A). The effect of the additional downstream sequence was greater on ACT1, reducing termination 5-fold while reducing termination of HBB1 only 2-fold (compare Figures 2B and 3A). This trend continued, with the addition of dsred spacer reducing termination in the ACT2 and ACT3 constructs approximately 3-fold, while the strongest terminator of the HBB series, HBB3 was affected less than 2-fold. In the presence of the downstream dsred spacer, HBB2 and HBB3 (both of which include the CoTC) are more effective than ACT2 and ACT3 respectively (Figure 3A) (p<0.05). This differential response to addition of dsred sequences downstream means that the effect of the dsred spacer cannot simply be attributed to adventitious promoters within the spacer. If transcription initiated within the dsred spacers to increase hRLUC expression, the effect would be expected to be highest in the constructs with the most repressed hRLUC such as HBB3, and lowest in the constructs with a fair amount of read-through to hRLUC such as ACT1 and HBB1.

To confirm that the luciferase reporters are representative of FLUC and hRLUC mRNA levels, and are not a reflection of changes in overall translation attributed to the addition of dsred sequence, we utilized real-time PCR to quantify luciferase mRNA (Figure 3B). Our data show the constructs reflect changes in FLUC and hRLUC mRNA levels, and support that our constructs quantify transcription elongation.

**Figure 4:**
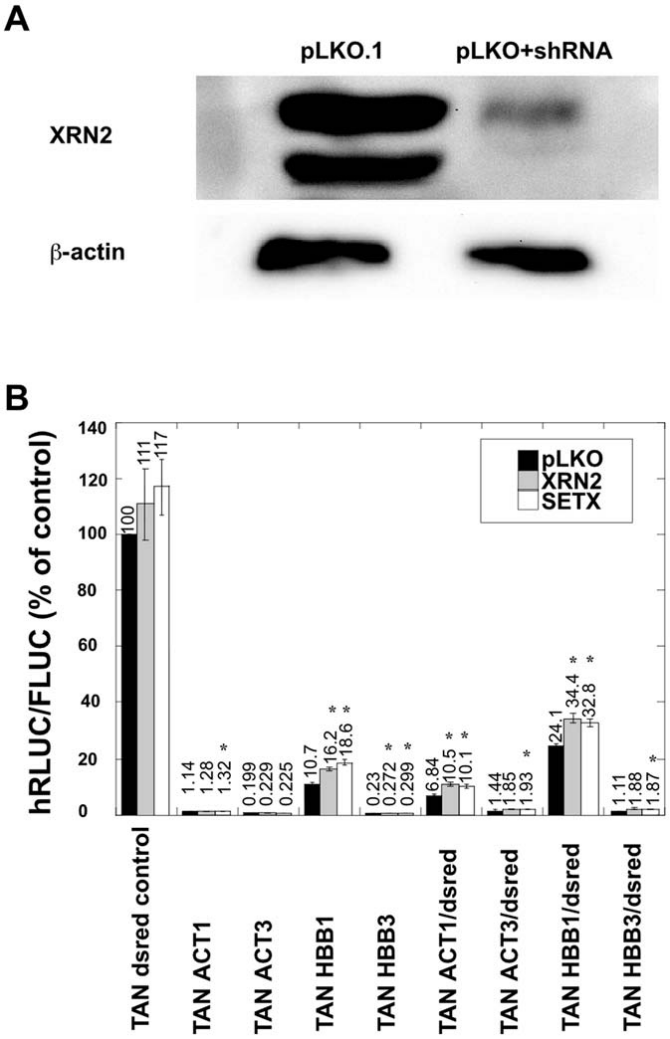
Xrn2 and Senataxin knockdowns provide limited release from transcription termination. *A*, Western blot analysis of Xrn2 knockdown in whole cell extracts. TAN dsred control cell line was grown as indicated in the methods and transfected with a plasmid expressing an shRNA targeting Xrn2. Samples were run on duplicate gels in parallel, blotted and then probed for Xrn2 expression. Anti-Xrn2 antibody (Bethyl Laboratories) produces a doublet, shown here. The lower band disappears with shRNA targeting Xrn2, and the upper band shows reduced density. Anti-human β-actin antibody serves as a control. The analysis was performed three times, with a representative blot shown. *B*, Xrn2 and senataxin knockdowns each provide little release from transcription termination. Cell lines were grown as detailed in the methods. The y-axis values are hRLUC/FLUC expression ratios normalized to a positive cell lysate run in each plate. Error bars indicate the S.E.M. for a sample number of three. Significant differences (p<0.05) from the corresponding vector (pLKO) treated samples are indicated by asterisks.

Transcription termination in our constructs is generally less effective with the addition of the dsred sequence downstream, counter to what we expected if the Xrn2 ‘torpedo’ model was a major contributor to termination. In order to determine to what degree Xrn2 contributed to termination in our system, we used shRNA to knock down Xrn2 and evaluated the effects in our cell lines. Knockdowns in our cell lines were done using a commercially available expression plasmid (pLKO), and insert sequences developed by the RNAi Consortium (TRC) and chosen from the NCBI probe website (for details, see methods). We performed western blots on protein extracts from cells treated with the empty vector pLKO.1 and with the shRNA-bearing vector to confirm shRNA-specific knockdown of Xrn2 (Figure 4A). Xrn2 was knocked down to similarly low levels in cell lines ACT1, ACT3, ACT1/dsred, ACT3/dsred, HBB1, HBB3, HBB1/dsred, and HBB3/dsred, and transcription elongation was quantified in Figure 4B.

Knockdown of Xrn2 in ACT1, ACT3, ACT3/dsred, and HBB3/dsred show no statistical difference from the control with regard to transcription elongation (p>0.05) (Figure 4B). Act1/dsred, HBB1, HBB1/dsred, and HBB3 show a minimal increase in transcription when Xrn2 is knocked down in the cell line (p<0.05) (Figure 4B).

While decreased Xrn2 does affect transcription elongation in some of our constructs, it provided only a modest increase in read-through, indicating a role for other proteins that are involved in transcription termination. Senataxin (Sen1) has considerable homology to Sen1p, a helicase essential for processing RNA in yeast [23], [26], and has been implicated in transcription regulation [27]. Consequently, senataxin is an excellent candidate for an alternative protein contributing to transcription termination in human cells. To test this, we knocked down senataxin levels in the same cell lines to evaluate the effect on transcription elongation (Figure 4B).

Senataxin levels were also knocked down using pLKO and TRC shRNA sequences. Our available antibodies to human senataxin did not prove to be reliable in western blot applications, so we confirmed knockdown of senataxin using real-time PCR. Quantitative PCR indicated that senataxin levels in the shRNA treatment were less than 20% of pLKO.1 treated cells (16±3%). Knockdown of senataxin in the cell lines increased transcription elongation in the absence of the dsred spacer sequence for poly(A) containing constructs ACT1, HBB1, and HBB3, but not ACT3 (Figure 4B). When a dsred spacer sequence is included, all four constructs show a significant (p<0.05) albeit small increase in transcription elongation.

In sum, we have developed a system capable of probing template-mediated influences on the elongation/termination decision by RNAP II. As a test of this system, we measured the termination associated with two well-characterized polyadenylation signals. We compared defined regions surrounding the human β-actin and β-globin polyadenylation sites and confirmed that even short tracts were effective at terminating transcription. Using shRNA we knocked down two candidate proteins involved in transcription termination but only saw modest effects. While we had thought that addition of a length of heterologous sequence downstream of the polyadenylation signals would increase termination, the reverse was true. In addition, the magnitude of the response to the downstream sequence was gene dependent, with the ACTB sequences showing a greater release from termination.

## Materials and Methods

### Materials

Restriction enzymes were from New England Biolabs (Ipswich, MA). All other chemicals were from Sigma unless otherwise stated. All tissue culture reagents were from Invitrogen (Carlsbad, CA) unless otherwise stated.

### Construction of Plasmids/Vectors

We assembled a tandem reporter construct in pcDNA5/FRT/TO (Invitrogen, Carlsbad, CA). The CMVIE promoter in pcDNA5/FRT/TO contains two tet-operator sequences at the start of transcription and works in conjunction with the constitutively expressed tetracycline repressor in the T-REx cell lines (Invitrogen). To insert our reporters downstream of the pcDNA5/FRT/TO promoter the plasmid was digested with HindIII & BamH1 and a HindIII & BamH1 fragment containing firefly luciferase (FLUC) from pGL3-control (Promega, Madison, WI) was inserted to make pcDN/FRT/FL. A 160 bp self-cleaving ribozyme sequence that we have used extensively [15], [16] was PCR amplified with primers that add SpeI & EcoRI sites 5′ and MfeI, BglII & XbaI sites 3′. Ribozyme PCR product cut with SpeI & BglII was ligated into XbaI & BamH1 digested pcDN/FRT/FL to create pcDN/FRT/FL/RZ. EcoRI & BglII cut ribozyme PCR product was inserted just 5′ of the IRES in pIRES2-EGFP (Clontech, Palo Alto, CA) digested with EcoRI & BamH1. The resulting 5′/ribozyme/IRES-3′ fragment was excised using NheI & NcoI (at ATG of IRES) and ligated into NheI & NcoI (partial) digested phRL-TK (Promega) that contains humanized renilla luciferase. An XhoI & XbaI digested 1720 bp fragment with this ribozyme/IRES/hRLUC sequence was then ligated into pcDN/FRT/FL/RZ digested with XhoI & XbaI to yield the pcDN/FRT/FL/RZ/RZ/IRES/hRL (or TAN0) plasmid. Unique NotI and XhoI sites remaining between the ribozymes were expanded to a polylinker (5′-NotI, NheI, BamH1, XmaI, XhoI-3′) with oligonucleotides to make TAN1. A control tandem vector with no ribozymes was made by cutting TAN0 with MfeI partial and EcoR1 partial dropping out both ribozymes, removing the polylinker and creating compatible sticky ends.

Coding sequence spacers: PCR was used to add XbaI to one side of a fragment of GFP coding sequence from pIRES2-EGFP (1695–1254 bp on the Clontech plasmid, Clontech.com) and the sites NotI, NheI, and BamH1 to the other side. This PCR product was cut with XbaI & BamH1 and inserted into pREX [28] cut with BglII & SpeI. PCR was used to add BamH1, XmaI and XhoI to one side of a 361 bp fragment of the chloramphenicol acetyltransferase (CAT) coding sequence from pSV2CAT (4969–4608 bp Genbank M77788) and XbaI to the other side. This CAT fragment PCR product and the plasmid with the GFP fragment were cut with BamH1 & XbaI and joined to make a pREX-GC. PCR was used to generate a 529 bp fragment of dsred coding region from pDsRed1-Mito (700–1229 on the Clontech plasmid, Clontech.com) adding BamH1 and XbaI to one side, and NheI to the other. PCR fragments cut with XbaI & NheI and BamH1 & NheI were mixed, ligated, cut again with BamH1 & NheI and gel purified. A 2158 bp dsred tetramer was ligated into a GFP-CAT fragment construct digested with Nhe1 and BamH1. The Dsred tetramer insert (with flanking GFP and CAT fragments) was PCR amplified and inserted into the polylinker region of the Tan1 construct. The non-human sequences (GFP/CAT) flanking the polylinker site serve as unique priming sites. The tetramer fragment serves as a size control spacer sequence in our constructs.

The polyadenylation regions of the human β−globin (HBB, NM_000518) and β−actin (ACTB, NM_001101) genes were isolated using PCR. A first round of PCR used primers generated by Primer3 [29] to amplify from genomic DNA samples. In our numbering scheme, the poly(A) addition site for each gene is numbered +1 and corresponds to base 5203272 on human chr 11 for HBB, and to base 5533305 on human chr 7 for ACTB, using the March, 2006 numbering. Primer sets were paired as follows, HBB-513F with HBB+2390R, and ACT-449F with ACT+1826R. The second round of PCR used the product generated by the first round of PCR as its template and used new primer sets which generated restriction enzyme sites (Nhe I and Not I) at the very ends of the product for cloning. HBB-200NotF primer was paired with each of the following HBB+200NheR, HBB+1100NheR and HBB+1800NheR to make a product of 400, 1300, and 2000 bp long, termed HBB1, HBB2 and HBB3 respectively. The ACT-200NotF primer was paired with ACT+200NheR, ACT+1100NheR and ACT+1800NheR (reverse) primers to generate a product of 400, 1300 and 2000 bp long, called ACT1, ACT2 and ACT3 respectively in this work.

A minimal poly(A) addition site (MniACT) taken from the core of the ACTB site was made by annealing the following two oligodeoxyribonucleotides:

MniACTpA1 GGCCagttgAATA**AA**agtgcacaccttaaaaatgaggccaagtgtgactttgtggtgtg

MniACTpA2 CTAGcacaccacaaagtcacacttggcctcatttttaaggtgtgcact**TT**TATTcaact

A version of this with the polyadenylation signal mutated was put together with the following two oligodeoxyribonucleotides:

Mni0ACTpA1 GGCCagttgAATA**TT**agtgcacaccttaaaaatgaggccaagtgtgactttgtggtgtg

Mni0ACTpA2 CTAGcacaccacaaagtcacacttggcctcatttttaaggtgtgcact**AA**TATTcaact

The poly(A) hexamer signal is in capitals, and the mutated bases are bolded.

### Cell Transfections and Establishment of Stable Cell Lines

In order to integrate our constructs into a stable cell line in a consistent chromosomal location, we utilized the Invitrogen Flp-In T-REx system as described previously [30]. All cell lines were maintained in Dulbecco's modified Eagle's medium (DMEM) high glucose and 10% FBS (Hyclone, Logan, UT). Cell lines were induced with 1 µg/ml doxycycline (Sigma) for 24 hours before use in the luciferase experiments.

### shRNA knockdown of SETX and XRN2

Cells were plated in a 24-well plate and transfected with 250 ng of the shRNA construct using lipofectamine 2000 (Invitrogen) as per the manufacturer's protocol. The shRNA constructs cloned into the pLKO.1 vector were from Open Biosystems.

XRN2 shRNA: TRCN0000049899 (NM_012255 mRNA)

SETX shRNA: TRCN0000051517 (NM_015046 mRNA)

Transfected cells were split into a 100 mm tissue culture dish 24 hours post-transfection and selected with 1 µg/ml of puromycin for 5 days. Knockdowns were confirmed using western blots and qRT-PCR for XRN2 and SETX respectively.

### Western Blot Analysis of XRN2

Cells were scraped and lysed in 2X Laemmli Buffer (20% glycerol, 2% SDS, 100 mM Tris (pH 6.8), fresh 125 mM DTT). XRN2 western blots were performed by resolving 150 µg of protein on an 8% SDS-PAGE gel (37.5∶1) (Bio-Rad Mini Protean™ system). The samples were transferred to Immobilon-P membrane (Millipore) using a Bio-Rad semi-dry transfer apparatus. Membranes were blocked for an hour at room temperature in 20% evaporated Carnation Milk/PBS mixture followed by overnight incubation at 4°C with the primary antibodies.

Rabbit anti-human XRN2 (Bethyl labs) and mouse anti-β-actin primary antibodies (Sigma) were used at 1∶250 and 1∶5000 dilutions respectively. Goat anti-rabbit HRP conjugated secondary antibody (Pierce) and goat anti-mouse HRP conjugated secondary antibody (Molecular probes) were used at a dilution of 1∶10000, followed by visualization using ECL Advance™ (Amersham). Images were obtained with the Kodak Gel Logic 440 Imaging system and analyzed with Kodak Molecular Imaging software (Version 4.0).

### Dual Luciferase Assay

The dual luciferase reagent (DLR) kit from Promega was used according to the manufacturer's directions as described previously [30].

### Real Time Reverse Transcription-PCR

Synthesis of cDNA to quantify FLUC and hRLUC expression in the TAN and TAN/dsred cell lines was done directly from cell lysates using the SuperScript™ III Cells Direct cDNA Synthesis System (Invitrogen). To quantify the senataxin knockdown, RNA was obtained using TRI-Reagent (Molecular Research Center, Inc.). First strand cDNA synthesis was done with 5 ng of RNA template using the Sidestep™ II QPCR cDNA synthesis kit (Stratagene).

The primer sets: FLUC sense aagattcaaagtgcgctgctggtg; FLUC antisense, ttgcctgatacctggcagatggaa; hRLUC sense, aatggctcatatcgcctcctggat; hRLUC antisense, tggacgatcgccttgatcttgtct; SETX sense, ctcaagacctgtgcctgtca; SETX antisense, ggttccgactacccaacaga; 18S rRna sense, attcgaacgtctgccctatca; 18S rRNA antisense, gtcacccgtggtcaccatg, were purchased from IDT (Coralville, IA) and used at a final concentration of 400 nM. The iQ SYBR Green Supermix was used according to the manufacturer's recommendations (Biorad, Hercules, CA). Cycling conditions were 10 min at 95°C, and then 40 cycles of 95°C for 30 sec, 55°C for 1 min and 72°C for 30 sec. Standard curves for the FLUC and hRLUC primer set were made with dilutions of the Tandem dsred tetramer control plasmid as described {[31]. Senataxin knockdowns were confirmed by the ΔΔCt method using 18S rRNA as an internal control [32], [33]. Data was analyzed using the Mx3000P software (version 2.0).
